# Supplementary material for: Multi-tract multi-symptom relationships in pediatric concussion
Source: eLife. 2022 May 17;11:e70450. doi: 10.7554/eLife.70450 (PMC9132577; doi:10.7554/eLife.70450)
Supplement: Supplementary file 1. [file elife-70450-supp1.docx]

**Table S1.** Table listing labels retained in the DKT+aseg parcellation.

| Label Number | Full Name |
| --- | --- |
| 10/49 | Left/Right Thalamus Proper |
| 11/50 | Left/Right Caudate |
| 12/51 | Left/Right Putamen |
| 13/52 | Left/Right Pallidum |
| 17/53 | Left/Right Hippocampus |
| 18/54 | Left/Right Amygdala |
| 26/58 | Left/Right Accumbens |
| 1002/2002 | Left/Right Caudal Anterior Cingulate |
| 1003/2003 | Left/Right Caudal Middle Frontal |
| 1005/2005 | Left/Right Cuneus |
| 1006/2006 | Left/Right Entorhinal |
| 1007/2007 | Left/Right Fusiform |
| 1008/2008 | Left/Right Inferior Parietal |
| 1009/2009 | Left/Right Inferior Temporal |
| 1010/2010 | Left/Right Isthmus Cingulate |
| 1011/2011 | Left/Right Lateral Occipital |
| 1012/2012 | Left/Right Lateral Orbitofrontal |
| 1013/2013 | Left/Right Lingual |
| 1014/2014 | Left/Right Medial Orbitofrontal |
| 1015/2015 | Left/Right Middle Temporal |
| 1016/2016 | Left/Right Parahippocampal |
| 1017/2017 | Left/Right Paracentral |
| 1018/2018 | Left/Right Pars Opercularis |
| 1019/2019 | Left/Right Pars Orbitalis |
| 1020/2020 | Left/Right Pars Triangularis |
| 1021/2021 | Left/Right Pericalcarine |
| 1022/2022 | Left/Right Post Central |
| 1023/2023 | Left/Right Posterior Cingulate |
| 1024/2024 | Left/Right Pre Central |
| 1025/2025 | Left/Right Precuneus |
| 1026/2026 | Left/Right Rostral Anterior Cingulate |
| 1027/2027 | Left/Right Rostral Middle Frontal |
| 1028/2028 | Left/Right Superior Frontal |
| 1029/2029 | Left/Right Superior Parietal |
| 1030/2030 | Left/Right Superior Temporal |
| 1031/2031 | Left/Right Supramarginal |
| 1034/2034 | Left/Right Transverse Temporal |
| 1035/2035 | Left/Right Insula |
