# Supplementary material for: Multi-tract multi-symptom relationships in pediatric concussion
Source: eLife. 2022 May 17;11:e70450. doi: 10.7554/eLife.70450 (PMC9132577; doi:10.7554/eLife.70450)
Supplement: Supplementary file 2. — Note: PLSc1: Microstructural complexity PLSc; PLSc2: Axonal Density PLSc. [file elife-70450-supp2.docx]

**Table S2.** Table listing *p*-values of correlations between the expression of all retained multi-tract connectivity features and the time since the latest injury.

| **Multi-tract connectivity feature** | **PLSc1** | **PLSc2** |
| --- | --- | --- |
| MCF1 | 0.832 |  |
| MCF2 | 0.819 | 0.124 |
| MCF3 | 0.718 | 0.206 |
| MCF5 | 0.195 | 0.204 |
| MCF7 | 0.952 |  |
| MCF8 | 0.757 | 0.497 |
| MCF9 | **0.048** | 0.182 |
| MCF10 | 0.951 | 0.951 |
| MCF11 | 0.299 | 0.349 |
| MCF12 | 0.067 |  |
| MCF13 | 0.524 |  |
| MCF14 | 0.299 |  |
| MCF15 | 0.242 |  |
| MCF16 | 0.524 |  |
| MCF17 | 0.332 |  |
| MCF18 | 0.897 |  |

PLSc1: Microstructural complexity PLSc; PLSc2: Axonal Density PLSc.
