# Supplementary material for: Multi-tract multi-symptom relationships in pediatric concussion
Source: eLife. 2022 May 17;11:e70450. doi: 10.7554/eLife.70450 (PMC9132577; doi:10.7554/eLife.70450)
Supplement: Supplementary file 3. — Note: PLSc1: Microstructural complexity PLSc; PLSc2: Axonal Density PLSc. [file elife-70450-supp3.docx]

**Table S3.** Table listing *p*-values of correlations between the expression of all retained multi-tract connectivity features and a variable indexing ADHD.

| **Multi-tract connectivity/Multi-symptom feature** | **Multi-tract connectivity feature** | | **Multi-tract symptom feature** | |
| --- | --- | --- | --- | --- |
|  | **PLSc1** | **PLSc2** | **PLSc1** | **PLSc2** |
| 1 | 0.003 |  | <0.001 |  |
| 2 | 0.835 | 0.957 | 0.064 | <0.001 |
| 3 | 0.228 | 0.367 | 0.005 | 0.196 |
| 5 | 0.643 | 0.169 | 0.029 | 0.027 |
| 7 | <0.001 |  | 0.008 |  |
| 8 | <0.001 | 0.092 | 0.002 | 0.112 |
| 9 | 0.754 | 0.174 | 0.546 | 0.933 |
| 10 | 0.236 | 0.012 | 0.169 | 0.195 |
| 11 | 0.066 | 0.743 | 0.135 | 0.284 |
| 12 | 0.567 |  | 0.212 |  |
| 13 | 0.878 |  | 0.408 |  |
| 14 | 0.304 |  | 0.220 |  |
| 15 | 0.333 |  | 0.827 |  |
| 16 | <0.001 |  | <0.001 |  |
| 17 | 0.425 |  | 0.576 |  |
| 18 | 0.782 |  | 0.646 |  |

PLSc1: Microstructural complexity PLSc; PLSc2: Axonal Density PLSc.
